# Evidence of altered mucosa-associated and fecal microbiota composition in patients with Irritable Bowel Syndrome

**DOI:** 10.1038/s41598-020-57468-y

**Published:** 2020-01-17

**Authors:** Johanna Sundin, Imran Aziz, Sofia Nordlander, Annikka Polster, Yue O. O. Hu, Luisa W. Hugerth, Alexandra A. L. Pennhag, Lars Engstrand, Hans Törnblom, Magnus Simrén, Lena Öhman

**Affiliations:** 1 Inst. of Medicine, University of Gothenburg, Gothenburg, Sweden; 2 Inst. of Biomedicine, University of Gothenburg, Gothenburg, Sweden; 3 Centre for Translational Microbiome Research (CTMR), Micobiology, Tumor and Cell Biology (MTC), Karolinska Institutet, 417164 Solna, Sweden; 4 Centre for Functional Gastrointestinal and Motility Disorders, University of North Carolina at Chapel Hill, Chapel Hill, NC USA

**Keywords:** Clinical microbiology, Dysbiosis

## Abstract

Altered bacterial composition and small intestinal bacterial overgrowth (SIBO) may be associated with irritable bowel syndrome (IBS). This study aimed to determine the fecal and mucosa-associated bacterial composition along the gastrointestinal (GI) tract and to assess SIBO in IBS. Bacterial composition of feces, and mucosa of the duodenum and sigmoid colon was determined by 16S rRNA-amplicon-sequencing. SIBO was evaluated by bacterial culture of duodenal aspirate, glucose and lactulose breath tests. Mucosal antibacterial gene expression was assessed by PCR Array. The bacterial profiles of feces and the mucosa of sigmoid colon, but not duodenum, differed between IBS patients (n = 17) and HS (n = 20). The IBS specific bacterial profiles were linked to the colonic antibacterial gene expression. Fecal bacterial profile differed between IBS subtypes, while the mucosa-associated bacterial profile was associated with IBS symptom severity and breath tests results at baseline (H_2_ and/or CH_4_ ≥ 15 ppm). The prevalence of SIBO was similar between IBS patients and HS. This study demonstrates that alterations in the bacterial composition of the sigmoid colon of IBS patients were linked to symptoms and immune activation. While breath tests reflected the mucosa-associated bacterial composition, there was no evidence for high prevalence of SIBO or small intestinal bacterial alterations in IBS.

## Introduction

Irritable bowel syndrome is a chronic functional bowel disorder which affects around 6% of the population^1^. The pathophysiology of IBS is multifactorial and has been suggested to include an altered bacterial composition of the gut. Additionally, small intestinal bacterial overgrowth (SIBO), characterized by abnormally high amounts of colonizing bacteria in the small bowel, has been suggested as an important feature in at least subsets of patients^2^.

Even though the reported prevalence of SIBO among IBS patients is highly variable, several studies, including a recent meta-analysis^2^, have shown a high prevalence of SIBO in IBS patients^3–5^. The current gold standard for diagnosing SIBO is bacterial culture of small intestinal aspirate^6^, but the majority of bacterial strains requires unknown growth conditions and have therefore not been cultured^7^, which could lead to false negative results. However, the majority of studies reporting high prevalence of SIBO in IBS patients are based on results from glucose and lactulose breath tests^2^, measuring the amount of hydrogen and methane produced by bacteria after digestion of ingested fermentable sugars, but do not include quantification of bacteria. The above mentioned discrepancies in evaluation methods most likely explain the conflicting reports of prevalence of SIBO reported in IBS^8^.

The majority of previous studies evaluating gut microbiota composition of IBS patients have focused on the fecal and colonic compartments. For example, previous results from our group showed a subset of IBS patients with an altered bacterial composition in feces^9,10^, and that the mucosa-associated bacterial composition of the colon in IBS patients was linked to mucosal low-grade immune activation^11,12^. However, so far only two previous studies have evaluated the small intestinal bacterial composition of IBS patients, demonstrating conflicting results^13,14^. To this date no studies have evaluated both the luminal and mucosa-associated bacterial composition in the upper and lower gastrointestinal tract in IBS patients.

In this study we hypothesized that IBS patients have an altered bacterial composition in the small and/or large intestine, which in turn is associated with the symptom pattern and the host´s intestinal immune response. Thus, the aim of this study was to determine the bacterial composition of fecal samples and biopsies from the small and large intestine, respectively, and to determine the link to symptom pattern and intestinal immune response in IBS patients. Further, this study aimed to explore the consistency between evaluation methods for SIBO and to explore the accordance between breath test results and intestinal bacterial composition.

## Results

### Clinical characteristics and demographics of IBS patients and healthy subjects

In total, 17 IBS patients and 20 healthy subjects were included in the study. Among patients, 2 were defined as IBS-C, 7 as IBS-D, 8 as IBS-nonCnonD (Table 1). Three patients were defined as having post-infectious IBS (PI-IBS) after a self-reported bout of gastroenteritis. There were no differences in age, BMI or gender between patients and healthy subjects (Table 1). As expected HADS and IBS-SSS scores were significantly higher in patients than in healthy subjects (Table 1).

**Table 1:** Clinical and Demographic factors of IBS patients and healthy subjects (HS).

	IBS	HS	*p*-value*
	(n = 17)	(n = 20)	
Sex (F/M)	12/5	9/11	*0*.*18*
Age (years)	30 (26–36)	26 (21–35)	*0*.*11*
BMI	22.4 (20.6–26.6)	22.0 (20.7–24.3)	*0*.*51*
HADS score^¤^	11 (6–20)	7 (3–10)	***0***.***008***
Anxiety^¤^	7	3	*0*.*14*
Depression^¤^	3	0	*0*.*09*
IBS-SSS score	277 (233–299)	11 (1–18)	<***0***.***0001***
Subtype (IBS-D/IBS-C/IBS-nonCnonD)	7/2/8	N/A	

Abbreviations:

IBS-C = Constipation predominant IBS. IBS-SSS = IBS Symptom Severity Score.

IBS-D = Diarrhea predominant IBS. HADS = Hospital Anxiety and Depression.

^¤^Based on HADS Anxiety ≥8, Based on HADS Depression >10.

Data shown as median (range 25–75%), Mann Whitney U Test between groups.

### Intestinal bacterial composition is highly dependent on sample type and regional localization

**Figure 1:**
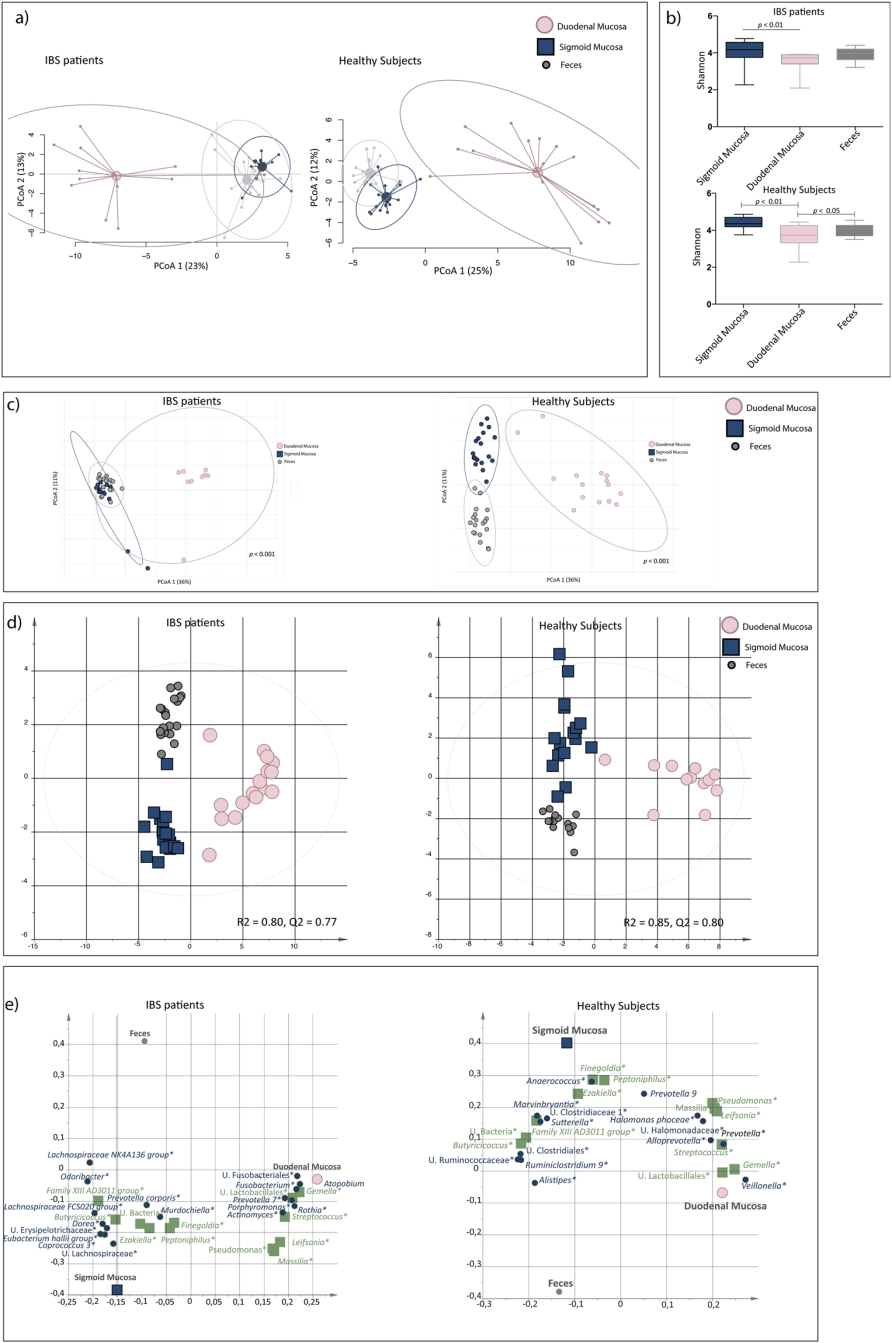
The fecal and mucosa-associated bacterial profiles of the small intestine and colon of IBS patients and healthy subjects. Profiles of the relative abundance of fecal bacteria (grey circles) and the mucosa-associated bacteria from the sigmoid colon (blue squares) and the duodenum (pink circles) of irritable bowel syndrome (IBS, n = 16) and healthy subjects (n = 19). (**a**) Principal component analysis (PCA) of IBS and healthy. (**b**) Shannon diversity of the bacterial composition of fecal samples and the mucosal-associated bacterial composition of the sigmoid colon and the duodenum of IBS and healthy. (**c**) Beta-diversity of the bacterial composition of fecal samples and the mucosa-associated bacterial composition of the sigmoid colon and the duodenum of IBS and healthy. (**d**) Multivariate orthogonal partial least squares-discriminant analysis (OPLS-DA) of IBS and healthy, respectively, with bacteria as X-variables and fecal samples, biopsies from the sigmoid colon and biopsies from the duodenum, respectively, as Y-variables. (**e**) Loading scatter plot from the OPLS-DA showing the separation between fecal samples and the mucosa-associated bacterial composition of the sigmoid colon and the duodenum, respectively, with the bacterial composition. Green squares represent bacteria which relative abundance contributed the most to the separation in the bacterial composition of the different sample types of both IBS and healthy, while blue circles are exclusively shown to contribute to the separation of the bacterial compositions of the different sample types of IBS or healthy, respectively. * = Significant difference between sample type according to the Kruskal-Wallis test. U. = Unclassified.

A principal component analysis (PCA) demonstrated that the general bacterial composition differed distinctly between samples obtained from feces, duodenum and the sigmoid colon, in IBS as well as in healthy (Fig. 1a). Shannon diversity (number of OTUs and evenness) was significantly lower in the duodenum compared to the sigmoid colon (*p* < 0.01) in both IBS and healthy (Fig. 1b), and the beta-diversity was structurally different between sample types and regional localization (*p* < 0.001) (Fig. 1c). The bacterial compositional difference of sample types and regional localization was further confirmed by orthogonal partial least squares-discriminant analysis (OPLS-DA) of IBS (R^2^ = 0.80, Q^2^ = 0.77) and healthy (R^2^ = 0.85, Q^2^ = 0.80) (Fig. 1d), respectively. The OPLS-DA also identified the bacteria (n = 26 and n = 29, respectively) that contributed the most to the separation among IBS and healthy (Fig. 1e). The majority of bacterial taxa driving the separation of bacterial composition between the sample type and regional localization were the same for IBS and healthy, whereas a few bacterial taxa were exclusive for IBS (Supplementary Table 3).

### Fecal luminal bacterial composition differ between IBS patients and healthy subjects

**Figure 2:**
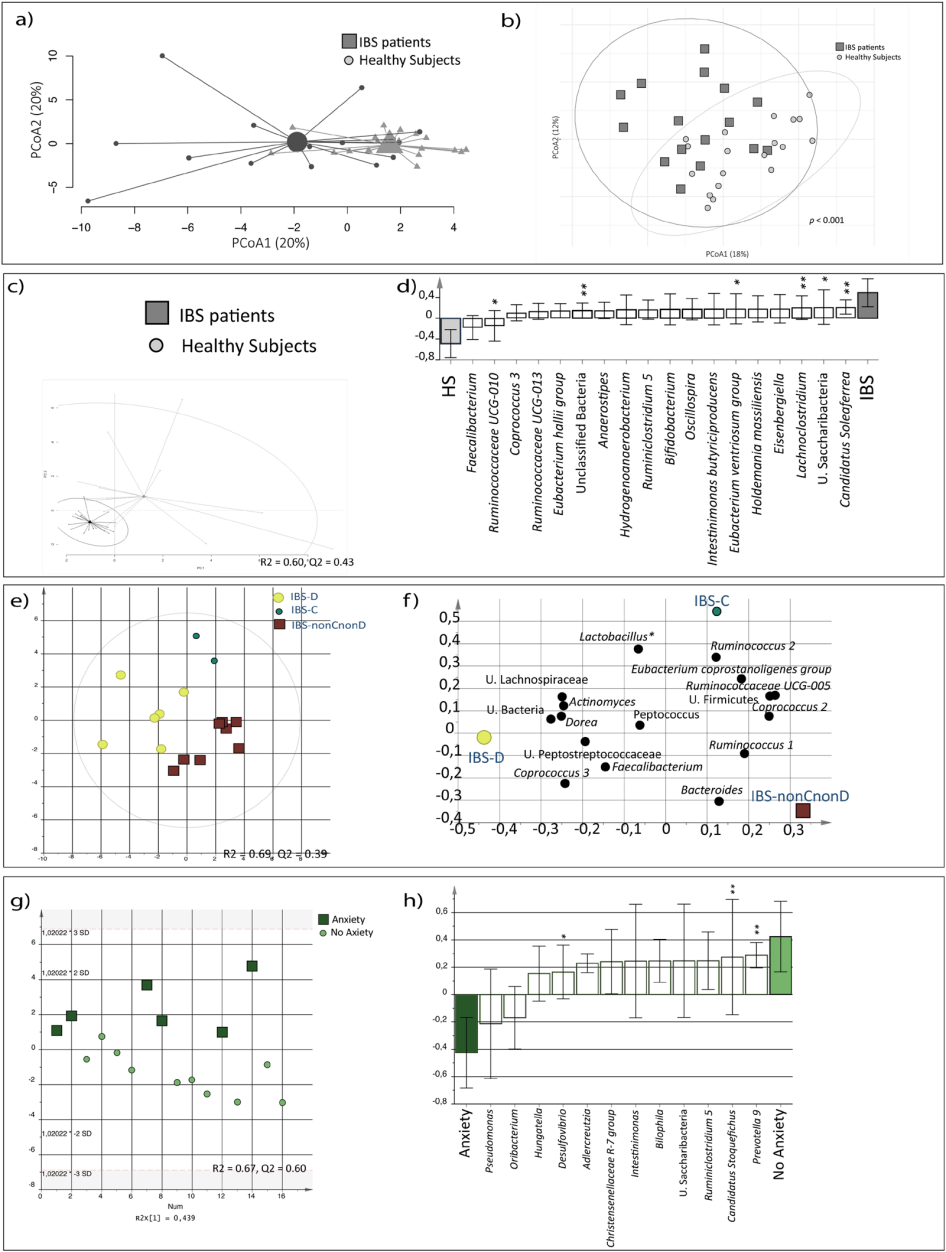
Fecal bacterial profiles of IBS patients and healthy subjects. Fecal bacterial composition of IBS patients (n = 16, dark grey squares) and healthy subjects (n = 19, light grey circles). (**a**) Principal component analysis (PCA) of IBS and healthy. (**b**) Beta-diversity analysis of IBS and healthy. (**c**) OPLS-DA showing the separation between IBS and healthy, respectively, and the fecal bacterial composition. Multivariate analysis was performed with fecal bacteria as X-variables and IBS and healthy, respectively, as Y-variables. (**d**) OPLS-DA loading column plot of the fecal bacteria that contributed the most to the separation between IBS and healthy. (**e**) Score scatter plot from the OPLS-DA showing the separation between the diarrhea predominant (IBS-D, n = 6; yellow large circle), constipation predominant (IBS-C, n = 2, green small circle) and the nonCnonD-IBS (n = 8, dark red square) subtypes, respectively, and the fecal bacterial composition. Multivariate analysis was performed with fecal bacteria as X-variables and IBS subtypes, respectively, as Y-variables. (**f**) OPLS-DA loading scatter plot of the fecal bacteria that contributed the most to the separation between IBS subtypes. **(g**) Score scatter plot from the OPLS-DA showing the separation between IBS patients with anxiety (n = 6; dark green squares), and without anxiety (n = 10, light green circles), respectively, and the fecal bacterial composition. Multivariate analysis was performed with fecal bacteria as X-variables and IBS patients with and without anxiety, respectively, as Y-variables. (**h**) OPLS-DA loading scatter plot of the fecal bacteria that contributed the most to the separation between IBS patients with and without anxiety. * = Significant difference between groups according to the Kruskal-Wallis test or the Mann-Whitney test. U. = Unclassified.

A PCA of the fecal bacterial composition did not show any major differences between IBS and healthy (Fig. 2a). However, beta-diversity differed between IBS and healthy (*p* < 0.001) (Fig. 2b). Further, an OPLS-DA (VIP > 1.4) demonstrated that the fecal bacterial profile of IBS differed from healthy (*p* = 0.016, R^2^ = 0.60, Q^2^ = 0.43), (Fig. 2c) and identified that the majority of bacteria (n = 18) that contributed the most to this separation, were found in higher relative abundance in IBS compared to healthy (Fig. 2d, Supplementary Table 4). Further, an OPLS-DA demonstrated different fecal bacterial compositions (R^2^ = 0.69, Q^2^ = 0.39) between IBS subtypes (IBS-D, IBS-C and IBS-nonCnonD), (Fig. 2e), and identified the bacteria (n = 16) that contributed the most to the separation. For instance, IBS-D patients had higher relative abundance of *Faecalibacterium* whereas IBS-nonCnonD patients had higher abundance of the *Bacteroides* (Fig. 2f). Additionally, patients with HAD anxiety scores ≥8 (n = 6), showed a different fecal bacterial composition (R^2^ = 0.67, Q^2^ = 0.60) compared to those with HAD anxiety scores <8 (Fig. 2g,h). Patients with severe IBS symptoms showed similar fecal bacterial profile compared to those with mild/moderate IBS symptoms (R^2^ = 0.43, Q^2^ = 0.19).

### The mucosa-associated bacterial composition of the sigmoid colon, but not duodenum, differ between IBS patients and healthy subjects

**Figure 3:**
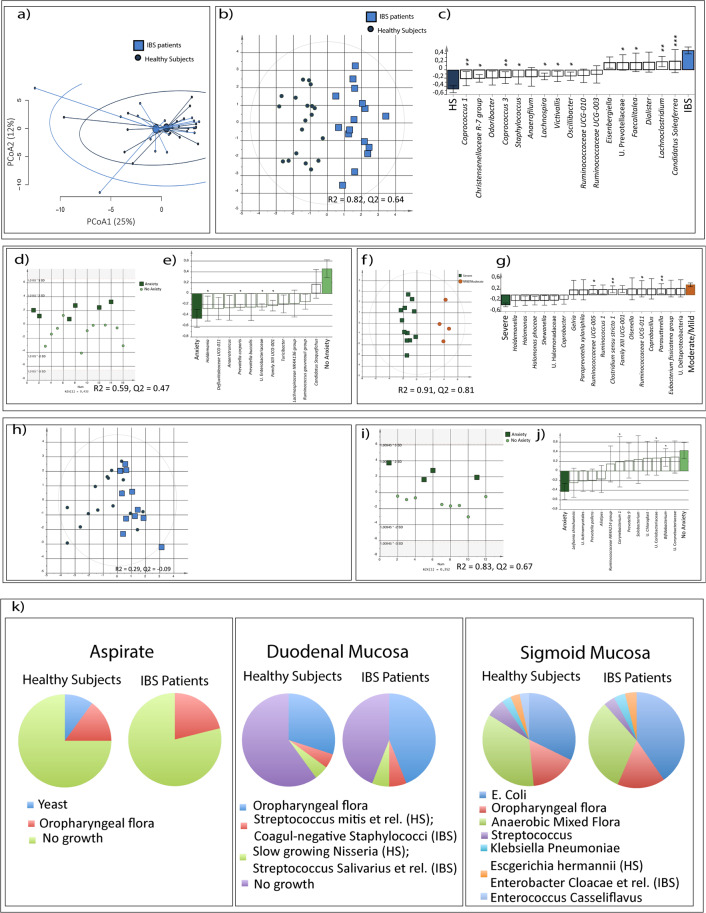
Mucosa-associated bacterial profiles and SIBO evaluation of IBS patients and healthy subjects. (**a**) PCA of the mucosa-associated bacterial composition of the colon of IBS and healthy. (**b**) Score scatter plot from the Multivariate orthogonal partial least squares-discriminant analysis (OPLS-DA) showing the separation between IBS (n = 16, light blue squares) and healthy (n = 19, dark blue cicles), respectively, and the mucosa-associated bacterial composition in the colon. **(c**) OPLS-DA loading column plot of the mucosa-associated bacteria of the colon that contributed the most to the separation between IBS and healthy. (**d**) Score scatter plot from the OPLS-DA showing the separation between IBS patients with anxiety (n = 6; dark green squares), and without anxiety (n = 10, light green circles), respectively, and the mucosa-associated bacterial composition in the colon. (**e**) OPLS-DA loading scatter plot of the mucosa-associated bacteria of the colon that contributed the most to the separation between IBS patients with and without anxiety. (**f**) Score scatter plot from the OPLS-DA showing the separation between symptom severity (IBS-SSS) based IBS subsets, mild/moderate IBS (IBS-SSS < 300, n = 4; orange circles) and severe IBS (IBS-SSS > 300, n = 12, green squares) respectively, and the mucosa-associated bacterial composition of the colon. (**g**) OPLS-DA loading column plot of the mucosa-associated bacteria of the colon that contributed the most to the separation between IBS patients with severe symptoms compared to those with mild/moderate symptoms. (**h**) OPLS-DA score scatter plot of the mucosa-associated bacterial composition of the duodenum of IBS (n = 12, light blue squares) compared to healthy (n = 19, dark blue circles). (**i**) OPLS-DA score scatter plot depicting the association between IBS patients with anxiety (n = 4; dark green squares), and without anxiety (n = 8, light green circles), respectively, and the mucosa-associated bacterial composition in the duodenum. (**j**) OPLS-DA loading scatter plot of the mucosa-associated bacteria of the duodenum that contributed the most to the separation between IBS patients with and without anxiety. (**k**) Proportions of bacterial growth in aspirates, duodenal biopsies and colonic biopsies, respectively in IBS (n = 16) and healthy (n = 19). * = Significant difference between groups according to the Mann-Whitney test or the Kruskal-Wallis test. U. = Unclassified.

Based on PCA there was no overall difference in the mucosa-associated bacterial composition of the sigmoid colon of IBS compared to healthy (Fig. 3a). However, OPLS-DA (VIP >1.4) demonstrated that the mucosa-associated bacterial composition of the sigmoid colon differed between IBS and healthy (*p* = 0.001, R^2^ = 0.82, Q^2^ = 0.64), and identified the bacterial genera (n = 17) that contributed the most to the separation (Fig. 3b). The loading column plot revealed lower relative abundance of *Coprococcus 1* and higher relative abundance of *Candidatus Soleaferrea* in IBS compared to healthy (Fig. 3c, Supplementary Table 3). According to OPLS-DA, the different IBS subtypes displayed similar mucosa-associated bacterial profiles of the sigmoid colon (R^2^ = 0.34, Q^2^ = 0.19), whereas patients with HAD anxiety scores ≥8 (n = 6), had a different bacterial composition of the sigmoid colon compared to those with HAD anxiety scores <8 (R^2^ = 0.59, Q^2^ = 0.47) (Fig. 3d,e). Additionally, patients with severe symptoms displayed a different bacterial profile compared to patients with mild/moderate symptoms (R^2^ = 0.91, Q^2^ = 0.81) (Fig. 3f). In general, the loading column plot display that the majority of the bacteria (n = 14) that contributed the most to the separation were found in higher relative abundance in patients with severe symptoms (Fig. 3g). In contrast, the mucosa-associated duodenal bacterial composition did not differ between IBS and healthy (OPLS-DA; VIP >1.4, *p* = 0.40, R^2^ = 0.29, Q^2^ = −0.09) (Fig. 3h, Supplementary Table 3) and no link to IBS subtypes or severity of IBS symptoms was demonstrated (OPLS-DA, VIP >1.4; R^2^ = 0.46, Q^2^ = −0.06 and R^2^ = 0.35, Q^2^ = 0.27, respectively). However, patients with HAD anxiety scores ≥ 8 (n = 4) displayed a different duodenal bacterial composition compared to those with HAD anxiety scores <8 (n = 8), (R^2^ = 0.83, Q^2^ = 0.67) (Fig. 3i,j).

### Bacterial cultures demonstrate similar prevalence of SIBO and bacterial growth in IBS patients and healthy subjects

None of the patients or healthy met the golden standard for SIBO ≥10^5^ CFU/ml anaerobic mixed bacterial flora^15^, neither in aspirate nor in duodenal biopsy cultures (Fig. 3k). Further, although there were individual variations in growth of opportunistic commensals, independent of sample type, there was no difference in bacterial growth between IBS and healthy (Fig. 3k).

### Breath tests demonstrate similar prevalence of proximal and distal SIBO of IBS patients and healthy subjects

**Figure 4:**
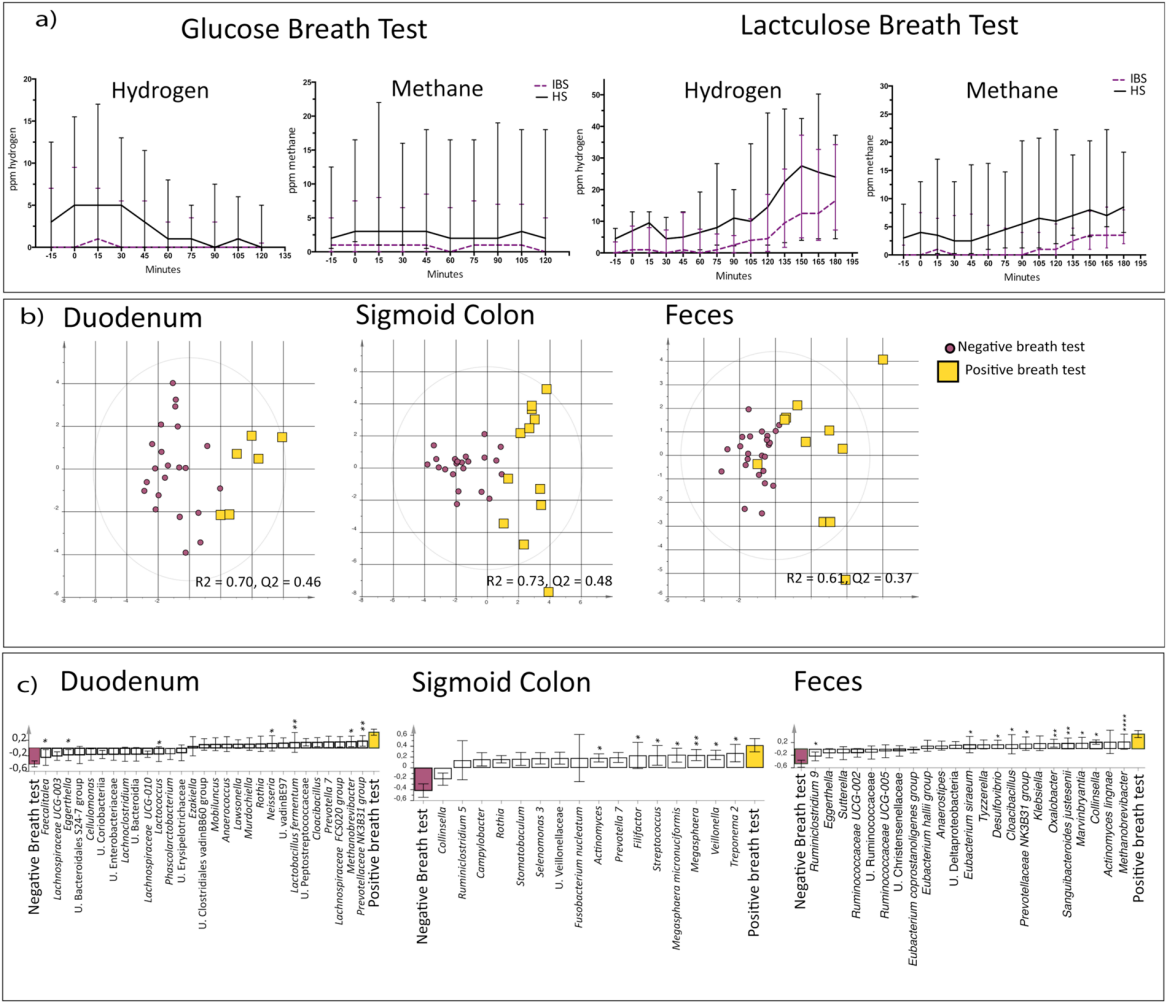
Glucose or lactulose breath tests and their link to the bacterial composition. (**a**) Exhaled H_2_ and CH_4_ over time, after glucose and lactulose ingestion in IBS (n = 17, purple dotted line) and healthy (n = 20, black solid line). (**b**) Multivariate orthogonal partial least squares-discriminant analysis (OPLS-DA) score scatter plot of the bacterial composition of feces, the mucosa-associated bacterial composition in the colon and the mucosa-associated bacterial composition in the duodenum of IBS and healthy with positive glucose or lactulose breath test (yellow squares) based on elevated baseline levels of H_2_ and/or CH_4_ (>15 ppm) compared to those with a negative test (purple circles). (**c**) OPLS-DA loading scatter plot showing the separation between IBS and healthy with positive fasting glucose or lactulose breath test compared to those with a negative test, and the bacterial composition of feces, the mucosa-associated bacterial composition in the colon and the mucosa-associated bacterial composition in the duodenum, respectively. Multivariate analysis was performed with bacteria as X-variables and IBS and healthy with a positive fasting breath test and those with a negative test at baseline, respectively, as Y-variables. * = Significant difference between those with a positive test compared to those with a negative test according to the Kruskal-Wallis test. U. = Unclassified.

Independent of breath test based definition the frequency of patients meeting the glucose and lactulose breath tests based SIBO criteria, indicating proximal and distal SIBO, respectively, was similar to healthy (Table 2). Moreover, when observed over time, no significant differences in neither H_2_ nor CH_4_, after glucose or lactulose intake were recorded between IBS and healthy (Fig. 4a) and IBS patients with high baseline values of either H_2_ or CH_4_ (262 (202–280)) displayed similar IBS symptom severity according to the IBS-SSS compared to patients with normal baseline values (285 (227–314)), (*p* = 0.32).

**Table 2:** IBS patients and healthy subjects (HS) with indication of small intestinal bacterial overgrowth according to different glucose and lactulose breath test based definitions.

	IBS (n = 17)	HS (n = 20)	*p*-value
**Glucose Breath test**			
Fasting levels of H_2_ ≥ 15 ppm	2	3	>0.99
Fasting levels of CH_4_ ≥ 15 ppm	3	5	0.71
Rise of H_2_ or CH_4_ ≥ 20 above baseline within 90 min	0	0	N.A.
**Lactulose Breath test**			
Fasting levels of H_2_ ≥ 15 ppm	1	3	0.61
Fasting levels of CH_4_ ≥ 15 ppm	1	3	0.61
Rise of H_2_ ≥ 20 ppm above baseline within 90 min	2	2	>0.99
Rise of CH_4_ ≥ 20 ppm above baseline within 90 min	1	0	0.44
Two distinct peaks of H_2_ or CH_4_ > 20 ppm	0	1	0.44

N.A. = Non applicable; HS = Healthy Subjects.

Investigation of potential links between H_2_ and CH_4_ levels and the bacterial composition were performed on individuals with a positive breath test using the baseline value (H_2_ and/or CH_4_ ≥ 15 ppm) (Table 2). When comparing individuals with a positive breath test (n = 12), (H_2_ and/or CH_4_ ≥ 15 ppm) and a negative breath test at baseline (n = 25) (Table 2), the bacterial composition differed between the two groups in the duodenal mucosa (R^2^ = 0.70, Q^2^ = 0.46) and sigmoid colonic mucosa (R^2^ = 0.73, Q^2^ = 0.48), but not in feces (R^2^ = 0.61, Q^2^ = 0.37) (Fig. 4b). The bacterial genera that contributed the most to the separation between the groups are shown in (Fig. 4c).

### Colonic antibacterial gene expression is linked to bacterial profiles of IBS patients

**Figure 5:**
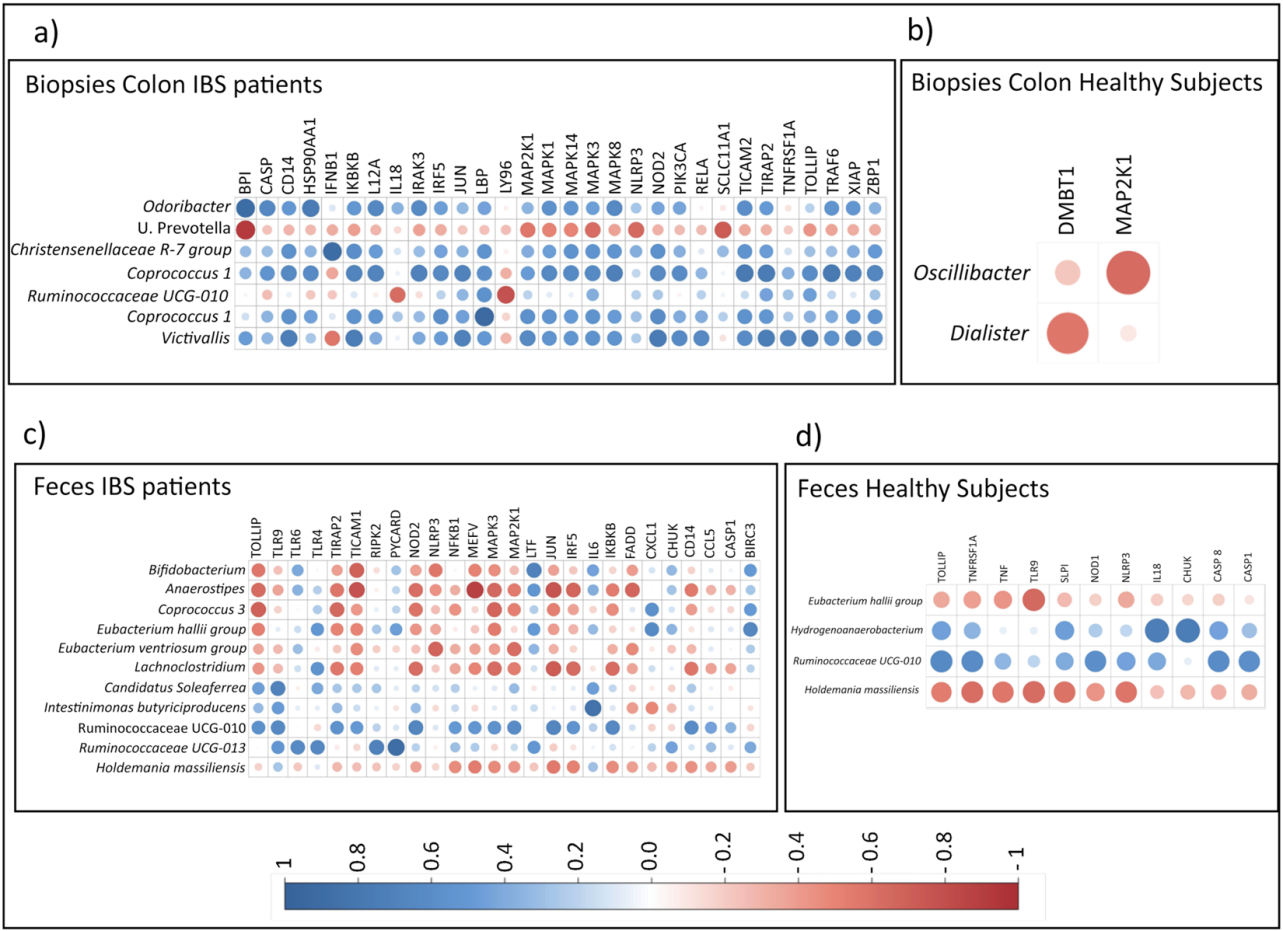
Clustered correlation matrix analysis between antibacterial gene expression and bacteria. Bacteria that were demonstrated to differentiate between IBS and healthy were included in clustered correlation matrix analysis between antibacterial gene expression and bacteria. Correlation between (**a**) antibacterial gene expression and the mucosa-associated bacteria of IBS patients, (**b**) antibacterial gene expression and the mucosa-associated bacteria of healthy subjects (**c**) antibacterial gene expression and fecal bacteria of IBS patients, (**d**) antibacterial gene expression and the fecal bacteria of healthy subjects, are shown. Positive correlations are displayed in blue and negative correlations in red. Color intensity and the size of the circle are proportional to the correlation coefficients, only significant correlations are shown (p < 0.01).

We further evaluated fecal and mucosa-associated bacteria of the colon in relation to the colonic immune response of the host. A clustered correlation matrix analysis of mucosal antimicrobial gene expression and the bacteria that contributed the most to the separation between IBS and healthy, and thus characterized the two groups were performed. Numerous correlations between antimicrobial gene expression and mucosa-associated as well as fecal bacteria were identified in IBS. In the colonic mucosa of IBS patients, the majority of the analyzed bacteria were positively correlated to the expression of many antimicrobial genes (n = 30) (Fig. 5a), and the fecal bacteria were either positively or negatively correlated to the expression of several antimicrobial genes (n = 21) (Fig. 5c). Substantially fewer correlations between the expression of antimicrobial genes (n = 2 and n = 11, respectively) and mucosa-associated or fecal bacteria were found in healthy (Fig. 5b,d).

## Discussion

This exploratory study demonstrated an altered bacterial composition in the colon of IBS patients compared to healthy subjects, where differences in both fecal and mucosal samples were detected. Additionally, anxiety, as well IBS subtype and IBS symptom severity were associated to intestinal bacterial composition. Further, a link between the IBS-specific colonic bacterial signature and expression of antibacterial genes was demonstrated. Despite lack of evidence supporting bacterial alterations in the small intestine or elevated prevalence of SIBO in IBS patients, elevated baseline values of breath methane and hydrogen were associated with the intestinal bacterial composition.

This is the first study making use of a broad range of analytical methods to comprehensively explore and compare small intestinal bacterial overgrowth (SIBO), using different definitions, with the mucosa-associated and luminal bacterial composition in both the small and large intestine of IBS patients and healthy subjects. The unique combination of sample types in this study, representing different regional localizations, allowed us to demonstrate that altered mucosa-associated and fecal bacterial composition specifically located in the colon in IBS patients. Contrary, both small intestinal bacterial composition and the prevalence of SIBO were similar in IBS patients compared to healthy subjects. This was the case even though presence of bacterial alterations in different parts of the gastrointestinal tract and SIBO were evaluated with several methods such as culture independent 16S analysis, bacterial cultures and glucose and lactulose breath tests. While all of the above mentioned methods have their limitations, the combination enhances the robustness of our results.

This study confirms that the global bacterial composition in different parts of the gastrointestinal tract in both IBS patients and healthy subjects are specific to sample type and regional location^16^. Moreover, our results suggest that bacteria of the mucosa and feces, respectively, could potentially be differently linked to IBS symptoms. Hence, fecal bacterial composition of IBS patients was linked to fecal stool form, which forms the basis for bowel habit-based IBS subtyping (IBS-D, IBS-C and IBS-nonCnonD)^17^ and thus confirm associations between stool consistency and bacterial composition previously shown in healthy subjects^18^. While the reasons for this link requires further investigation, one potential explanation could be that delayed transit is associated with enhanced proteolytic bacterial fermentation of proteins, associated with the formation of microbial cell components^19^. Moreover, the mucosa-associated bacteria, which are in close contact to host nerves and immune cells, were linked to IBS symptom severity. Potentially, this is a result of bacteria produced products that due to close proximity affect epithelial cells and trigger visceral pain responses either directly or through immune activation^20,21^. For instance, more severe symptoms were linked to reduced relative abundance of the Ruminococcaceae UCG-005 genera, previously shown to be associated with a healthy intestine^22^. Additionally, as previously shown in feces^11^, anxiety in IBS was associated with the composition of the mucosa-associated and fecal bacteria.

The high resolution of the bacterial analyses together with discriminant analyses (OPLS-DA) in our study allowed identification of signatures of fecal as well as sigmoid mucosa-associated bacteria, specific for IBS patients. A feature of the fecal and mucosa-associated sigmoid bacterial signature of IBS patients was reduced relative abundance of bacteria belonging to the Ruminococcaceae family. Reduction of these bacteria has previously been associated with aberrant immune activation, such as exaggerated Toll-like receptor 2 (TLR-2) responses^23^ and reduced production of short chain fatty acids (SCFAs) which improve barrier integrity and has anti-inflammatory properties^24^. Therefore, the low relative abundance of bacterial genera belonging to Ruminococcaceae shown in feces and in the sigmoid mucosa in IBS patients in this study suggests a potential underlying mechanism for the low grade inflammation previously shown in IBS^25,26^. Further, we confirmed previous reports of a subset of IBS patients with a different global fecal bacterial composition compared both to healthy subjects and to the remaining IBS patients^9–11^, emphasizing the importance to take the heterogeneity among IBS patients into account.

To ensure comprehensive investigation of SIBO, we followed the European and North American consensus reports for evaluation of SIBO with breath tests^27,28^, and performed bacterial cultures of aspirate according to the current golden standard^15^, but additionally evaluated the full bacterial composition of the small intestine of IBS patients with culture independent 16S analysis. Contrary to some recent studies^2,13^, we could not detect any evidence for altered small intestinal bacterial composition or increased prevalence of SIBO in IBS, independent of definition of SIBO. Potentially, the high prevalence of SIBO in previous reports may be a result of the fact that the majority of studies are based on breath tests^2^, which is an indirect test with numerous sources of error^29^. However, this exploratory study demonstrated a link between baseline concentration of hydrogen and methane measured with breath tests and the mucosa-associated bacterial composition of the duodenum and sigmoid colon, but not feces. As mucus-derived components provides an energy source for mucolytic bacteria, often present in close contact to the mucosa^30^, the baseline results of the breath tests could potentially be a result of diversity between individuals regarding the composition of mucosal glycans or the mucolytic bacteria that ferment mucosal glycans.

In line with a previous study from our group^12^, this study confirms that there are links between the fecal and mucosa-associated bacterial profiles of IBS and the colonic expression of antibacterial genes. In this study the bacteria most important for characterizing IBS patients were associated to the gene expression of numerous antimicrobial genes, whereas only few associations were seen in the corresponding analyses of healthy subjects. However, our data do not allow us to conclude whether the altered bacterial composition is a cause or a consequence of the host response, although it seem rather clear that these parameters are linked in the IBS patients, and possibly resulting in, or reflecting, a destabilized intestinal microenvironment.

One of the limitations of this study is it’s the cross-sectional nature, since it is well known that intestinal bacterial composition fluctuates over time^31–33^. However, this limitation is also true for the majority of studies of the bacterial composition and more importantly also for clinical investigations of SIBO in IBS^9–11,34^. Further, the relatively small study cohort limits the strength of the conclusions to be drawn from the study. All study procedures were strictly standardized and the same researchers performed sample collection during all endoscopies, in order to limit variability of bacterial composition due to differences in sample collection routines. Furthermore, the mucosa-associated bacteria composition of the duodenum is based on a relatively low number of sample reads, which most likely reflects the low bacterial load in this part of the small intestine^31^. However, to ensure that this did not have any major influence on the results, all analyses were performed with cut-offs of 1000 and 500 reads, respectively, and there were no major differences in the results between these analyses. Another limitation is that patients were not included based on the updated ROME IV criteria, but on the ROME III criteria, making comparisons between this study and studies selecting patients according to the ROME IV criteria somewhat difficult. However, a study on a corresponding Swedish cohort has shown that most ROME III-positive IBS patients seeking healthcare fulfil the ROME IV criteria^35^.

To conclude, the findings of this unique study, evaluating both sample type and regional localization specific bacterial composition alterations using several different methods, demonstrated alterations in the bacterial composition in the colon, but not in the small intestine of patients with IBS. Despite lack of evidence in this study for high prevalence of SIBO or small intestinal bacterial alterations of IBS patients, breath test results reflected the intestinal bacterial composition. Further, this study demonstrated that bacterial alterations of the colon are associated to the intestinal immune response and also to psychological and gastrointestinal symptoms, indicating that the bacterial composition has a central role in IBS pathophysiology.

## Method

### Study population and material sampling

IBS patients who met the ROME III diagnostic criteria^36^ were recruited from the outpatient clinic at Sahlgrenska University Hospital Gothenburg, Sweden. Exclusion criteria included microscopic colitis, celiac disease, food allergies, or any other GI disease explaining the symptoms. Other exclusion criteria included abnormal results on standard screening laboratory tests, severe psychiatric, systemic or other chronic diseases, history of drug or alcohol abuse, and the inability to reliably respond to questionnaires in Swedish. Based on recorded bowel movements in a one-week stool diary, IBS patients were subtyped into diarrhea-predominant IBS (IBS-D), constipation-predominant IBS (IBS-C), while IBS patients with mixed loose and hard stools (IBS-M) and those who had unsubtyped IBS (IBS-U) were grouped together (non-constipation-non-diarrhea predominant, nonCnonD)^36^. Healthy subjects without gastrointestinal (GI) symptoms (assessed for seven days prior to inclusion with a GI symptom screening questionnaire), psychiatric, gastrointestinal, cardiac or metabolic diseases were included. IBS patients and healthy subjects were of Caucasian ethnic origin. Informed consent was obtained from all participants before any study-related procedures. We recorded the use of specific diets and medications in all study subjects (Supplementary Table 1).

From all study subjects, biopsies were obtained from the second part of the duodenum and the sigmoid colon (25–35 cm proximal from the anus) by the same gastroenterologist (IA) using standard biopsy forceps without prior bowel preparation. Once collected all samples were handled by one researcher (JS), biopsies were immediately snap frozen in liquid nitrogen and stored at −80 °C until further analysis or placed in NaCl 0.9% solution and transferred within 2 h to microbiology for culture. Duodenal juice was aspirated prior to biopsy collection from participants where this was possible, i.e. where intestinal liquid was present and could be aspirated through the biopsy channel of the endoscope and collected in a sterile, DNA/RNA free, plastic tube and transferred within 2 h to microbiology for culture. Fecal samples were collected by patients at home, immediately frozen in −20 °C and brought frozen to the laboratory where it was stored frozen in −80 °C until analysis. The study was approved by the Regional Ethical review Board in Gothenburg, and all subjects provided informed consent before any study related procedures were initiated. All experiments were performed in accordance with relevant guidelines and regulations.

### Bacterial cultures

Cultures of aerobic and anaerobic bacteria were made on blood agar plates with 4% defibrinated horse blood in aerobic and anaerobic atmospheres of N_2_ and 10% CO_2_ at 37 °C. Selective cultivation of Gram-negative strains was performed on Drigalski agar under aerobic conditions. Yeast fungus was cultured on Sabouraud’s agar. The minimum incubation time was 48 h. Identification of the microorganisms was based on colony characteristics, Gram staining, biochemical and chromatographic tests and Maldi-tof (Vitek-MS, Biomerieux). Quantification was performed by counting the number of colony-forming units (cfu/ml). Culture-verified SIBO was defined as >10^5^ cfu/ml of colonic bacteria according to the current golden standard^15^.

### Glucose and lactulose breath tests

Breath tests were performed after at least one day with low fiber diet, after fasting overnight, a short resting period in the morning and after careful brushing of the teeth. In addition, the subject was instructed not to consume any laxatives, bulking agents, or bile acid sequestrants the day before the test. If the first baseline value exceeded 15 parts per million (ppm) for methane or hydrogen, the subject performed a mouthwash with tap water before the next baseline value was collected. If the subject had repeatedly high baseline values the subject was rescheduled for an examination another day, after even more stringent instructions about preparation for the test, please see above, and after 24 hours of fiber-free diet. Subjects with high baseline values were rescheduled for a maximum of three visits. After recording two baseline values, subjects ingested 45 gram of glucose (Apoteket Produktion & Laboratorier, Sweden) in 300 ml tap water, or lactulose in the form of syrup (15 mL of syrup solution 670 mg/mL) (Laktulos Meda, Meda AB, Solna, Sweden), respectively. Hydrogen and methane gas in expired air was measured every 15 minutes during the following 120 (glucose) or 180 (lactulose) minutes. The expired air was collected in a two-bag system (Quintron, Milwaukee, WI, USA) with a valve and mouthpiece. The exhaled air was then analyzed with a breath gas analyzer (BreathTracker, Digital microlyzer; Quintron, Milwaukee, WI, USA).

The breath test based definitions of SIBO were based on either hydrogen or methane gas, respectively. The criteria were categorized into: (1) fasting levels (abbreviated fasting criteria of H_2_ or CH_4_ ≥ 15 ppm^37,38^; (2) a rise of H_2_ or CH_4_ above baseline within 90 min (abbreviated increase criteria ≥20 ppm)^4,28,39^; (3) for the lactulose breath test occurrence of double peaks (two distinct peaks or a characteristic double peak pattern >20 ppm on two consecutive readings^40^. The criteria for SIBO based on fasting breath levels were evaluated on the basis of the mean of the subject’s baseline values.

### Microbiota composition assessment in fecal and mucosal samples

DNA was extracted as previously described^41^ from feces (n = 35), duodenal biopsies (n = 28), sigmoid colonic biopsies (n = 35). In short, 1-step PCR procedure, amplification was carried out by a high fidelity proofreading polymerase for a total of 25 cycles (fecal samples) or 33 cycles (biopsies). For amplification of the sequencing libraries, forward primer 5′-CAAGCAGAAGACGGCATACGAGAT-N8-GTCTCGTGGGCTCGGAGATGTGTATAAGAGACAGGACTACHVGGGTATCTAATCC-3′ and reverse primer 5′AATGATACGGCGACCACCGAGATC-N8-TCGTCGGCAGCGTCAGATGTGTATAAGAGACAGCCTACGGGNGGCWGCAG-3′, where N8 represents an identifying 8-mer and the last 21 and 19 bases in each construct are the sequence specific forward and reverse primers, respectively. Samples were then pooled to equimolar amounts and sequenced in parallel to whole bacterial genomes in a MiSeq instrument (Illumina Inc, San Diego, CA, USA). All controls from the extraction phase, as well as a negative PCR control have been submitted to PCR and consequently sequenced with the respective samples. Raw reads quality filtering and trimming, operational taxonomic units (OTU) clustering- and taxonomic assignment were performed as previously described^41^. α-diversity was evaluated on Shannon index and number of OTUs following rarefaction at 1099 sequences using the vegan R package. Beta-diversity (diversity between samples) was performed on Bray-Curtis index. Samples exceeding 10000 number of reads for fecal samples, 500 for duodenal biopsies and 15000 for colonic biopsies (Supplementary Table 2) were used. To ensure that the low number of reads did not impact the results from the duodenum, all analysis were additionally performed with only samples exceeding 1000 reads, which gave similar results in all analysis.

### Questionnaires

Subjects completed a HADS and IBS-SSS to assess IBS and psychological symptom severity; for further details please see Supplementary Material.

### Antibacterial gene expression profile analysis

Human Antibacterial Response RT² Profiler PCR Arrays (Cat No.ID PAHS-148Z, Qiagen) were conducted as previously described^12^, on biopsies from duodenum and colon. For further details please see Supplementary Material.

### Data processing and statistical analysis

#### Univariate analysis

Univariate analysis was performed in GraphPad Prism 6 (GraphPad Software, La Jolla, CA), Mann-Whitney was used to assess differences between two groups and Kruskal-Wallis followed by Dunn’s test, was implemented for differences between three groups. To evaluate differences between the slopes of the curves of hydrogen and methane production over time in IBS patients and healthy subjects, linear regression analyses were performed on breath test data. If no other specification, results are presented as median ± (25–75% percentile). Group differences in beta-diversity were determined with PERMANOVA. Correlations between bacteria and antibacterial factors were analyzed with non-parametric Spearman correlations.

#### Multivariate analysis

Principal component analysis (PCA) was performed in R (Version 1.0.153) in the package ropls, to explore potential differences in the global bacterial and antibacterial recognition factor profiles of groups. Clustered correlation matrix analysis was performed on gene expression data using the corrplot-package in R, calculations were performed with Spearman’s Rank Correlation Coefficient.

Multivariate factor orthogonal partial least squares-discriminant analysis (OPLS-DA) was performed to determine the most discriminatory X variables and to discriminate between healthy subjects and IBS patients as well as for IBS patients clustered into subtypes and subjects positive/negative for SIBO according to glucose or lactulose breath tests, using SIMCA software (Version 14.1.3.0, copyright © MKS Data Analytics Solutions) based on bacterial composition (X variables). The R^2^ parameter represents the goodness of the fit of the OPLS-DA with the best possible fit being R^2^ = 1. When considering heterogeneous biological variables, a model would be considered to have a good fit with an R^2^ ≥ 0.5. The Q^2^ parameter represents an estimate of the predictive ability calculated by cross-validation, with the best possible predictive ability of the OPLS-DA being Q^2^ = 1, a Q^2^ value > 0.4 is considered good for biological variables^42^. In multivariate analysis, outliers were removed if they were both above the Hotelling’s T^2^ Range Line of 95% and DModX DCrit (0.05). Loading column plots were generated to identify the X-variables most important for the discrimination between subsets. Variable Importance for the Projection (VIP) > 1.4 was used as the number of bacterial genera exceeded the number of subjects by far, except when identifying the bacterial genera that contributed most to the underlying variation in the X variables (bacterial genera) between the different IBS subtypes, few bacterial genera reach a VIP over 1.4, therefore VIP > 1.1 was used for these analysis. To reduce the risk of over-fit post-hoc 100 permutation tests of the OPLS-DA models were performed and only models with permutation indices fulfilling the post-hoc analysis criteria of R^2^ < 0.4 and Q^2^ < 0.05 were accepted^43^. The loading scatter plot may be superimposed over the OPLS-DA score scatter plot to see what X-variables are associated with which individuals. X variables localized further away from the center of the x-axis contribute more to the discrimination of the groups. The results are presented as a score scatter plot, a loading scatter plot or a loading column plot in which the stronger variables with a more reliable contribution to the model are those with a large column and small confidence interval. Multivariate differences of X variables of the OPLS-DA, between patients and healthy subjects, were tested for significance using Hotelling’s T2 test in R (Package “Hotelling”).

## Supplementary information

Supplementary Information
