# Supplementary material for: Evidence of altered mucosa-associated and fecal microbiota composition in patients with Irritable Bowel Syndrome
Source: Sci Rep. 2020 Jan 17;10:593. doi: 10.1038/s41598-020-57468-y (PMC6969101; doi:10.1038/s41598-020-57468-y)
Supplement: Supplementary file 1 — Supplementary Information [file 41598_2020_57468_MOESM1_ESM.docx]

# Evidence of altered mucosa-associated and fecal microbiota composition in patients with Irritable Bowel Syndrome

**Johanna Sundin, Imran Aziz, Sofia Nordlander, Annikka Polster, Yue O.O. Hu, Luisa W. Hugerth, Alexandra AL Pennhag, Lars Engstrand, Hans Törnblom, Magnus Simrén, Lena Öhman.**

**Supplementary Methods**

**Study population and material sampling**

Biopsies were obtained from the second part of the duodenum and the sigmoid colon (25-35 cm proximal from the anus) using standard biopsy forceps without prior bowel preparation. Biopsies were immediately frozen in liquid nitrogen and stored at -80°C or placed in 0.9%NaCl for culture. Duodenal juice was aspirated to a sterile, DNA/RNA free, plastic tube prior to biopsy collection. Fecal samples were collected by subjects at home, immediately frozen in -20°C and brought frozen to the laboratory where it was stored frozen in -80°C.

**Bacterial cultures**

Cultures of bacteria was made on blood agar plates with 4% defibrinated horse blood in aerobic and anaerobic atmospheres of N_2_ and 10% CO_2_ at 37°C. Selective cultivation of Gram-negative strains was performed on Drigalskiagar under aerobic conditions. Fungus was cultured on Sabouraud’s agar. The minimum incubation time was 48h. Identification of the microorganisms was based on colony characteristics, Gram staining, biochemical and chromatographic tests and Maldi-tof (Vitek-MS, Biomerieux).

**Antibacterial gene expression profile analysis**

To profile the expression of 84 key genes involved in innate immune response to microbes. Briefly, each biopsy was placed in lysis buffer and homogenized for two bursts lasting two minutes following manufacturer’s protocol (Ref. 740955.50, Macherey-Nagel, Düren, Germany). Messenger RNA (mRNA) was extracted with the NucleoSpin® RNA Kit and stored at -80°C. The mRNA was then reverse transcribed creating cDNA using “RT² First Strand Kit” (Cat No.ID 330401, Qiagen). According to manufacturers’ protocol, cDNA together with RT² SYBR® Green qPCR Mastermix (Cat No.ID 330501, Qiagen) was transferred to the wells of the PCR array, which were analyzed using a Bio-Rad iCycler (Bio-Rad Laboratories, Inc. USA). All samples were done in duplicate and passed the quality checks for PCR Array reproducibility, RT efficiency and genomic DNA contamination. ACTB, GAPDH and HPRT were chosen as reference housekeeping genes. Their average expression was used to normalize the expression of the targeted gene sequence, whereby the difference between the average expression of reference genes and that of the gene targets is given as ΔCt (cycle threshold) and presented as 2−(Target-Reference) or (2ΔCt).

**Microbiota composition assessment in fecal and mucosal samples**

For amplification of the sequencing libraries, forward primer 5’-CAAGCAGAAGACGGCATACGAGAT-N8-GTCTCGTGGGCTCGGAGATGTGTATAAGAGACAGGACTACHVGGGTATCTAATCC-3’ and reverse primer 5´AATGATACGGCGACCACCGAGATC-N8-TCGTCGGCAGCGTCAGATGTGTATAAGAGACAGCCTACGGGNGGCWGCAG-3’, where N8 represents an identifying 8-mer and the last 21 and 19 bases in each construct are the sequence specific forward and reverse primers, respectively. Samples were then pooled to equimolar amounts and sequenced in parallel to whole bacterial genomes in a MiSeq instrument (Illumina Inc, San Diego, CA, USA). All controls from the extraction phase, as well as a negative PCR control have been submitted to PCR and consequently sequenced with the respective samples. To ensure that the low number of reads did not impact the results from the duodenum, all analysis were additionally performed with only samples exceeding 1000 reads, which gave similar results in all analysis.

**Questionnaires**

*The IBS Severity Scoring System (IBS-SSS)*

IBS-SSS, which includes five items (pain severity, pain frequency, severity of abdominal distension, bowel habit dissatisfaction and daily life interference), was used to assess IBS symptom severity. The scores are combined into an overall IBS score ranging from 0 (no symptoms) to 500 (maximum severity) ^23^. We used the total score of this questionnaire to analyze the severity of IBS. Patients with a total IBS-SSS score ≤ 300 were defined as having mild/moderate symptoms, while patients with a total IBS-SSS score of ≥ 300 were defined as having severe symptoms.

*Hospital anxiety and depression (HAD)*

To detect the severity of anxiety and depression and measure psychological distress, IBS patients and healthy subjects answered the 14-questions questionnaire HADS, using a four point Likert scale ranging from 0-3. Seven questions relate to anxiety and seven to depression with a minimum and maximum score of zero and three respectively with a high score indicating more severe symptoms ^24^.

**Supplementary Tables**

Table S1. Diet and medications of IBS patients and healthy subjects (HS).

|  | **IBS** | **Healthy** | **p-value** |
| --- | --- | --- | --- |
| **Diet** |  |  |  |
| No dietary restrictions | 9/17 | 17/20 | 0.07 |
| Low FODMAP | 2/17 | 0/20 | 0.20 |
| Lactose free | 3/17 | 0/20 | 0.09 |
| Semivegetarian | 1/17 | 1/20 | >0.99 |
| Vegetarian | 1/17 | 2/20 | >0.99 |
| Gluten free | 3/17 | 0/20 | 0.09 |
| **Medication** |  |  |  |
| SSRI | 5/17 | 0/20 | 0.01 |
| Proton pump inhibitor | 1/17 | 0/20 | 0.46 |
| Herbal product | 1/17 | 0/20 | 0.46 |
| Beta blocking agent (non-IBS indication) | 1/17 | 0/20 | 0.46 |

SSRI = Selective serotonin reuptake inhibitor

FODMAP= Fermentable Oligo-, Di-, Mono-saccharides And Polyols

Table S2. *Number of reads per sample for fecal samples, duodenal colonic biopsies and sigmoidal small intestinal biopsies.*

| **Sample** | **Number of reads** |
| --- | --- |
| **Biopsy Colon** |  |
| TIBS001 | 4392 |
| TIBS002 | 33182 |
| TIBS004 | 26926 |
| TIBS005 | 14357 |
| TIBS008 | 30714 |
| TIBS009 | 36369 |
| TIBS012 | 36492 |
| TIBS013 | 37647 |
| TIBS014 | 32048 |
| TIBS015 | 43879 |
| TIBS016 | 37106 |
| TIBS017 | 29894 |
| TIBS018 | 11606 |
| TIBS019 | 28292 |
| TIBS020 | 32199 |
| TIBS021 | 8382 |
| TIBS023 | 20677 |
| TIBS024 | 25883 |
| TIBS025 | 28982 |
| TIBS026 | 27914 |
| TIBS027 | 28589 |
| TIBS028 | 36879 |
| TIBS029 | 30514 |
| TIBS030 | 26060 |
| TIBS032 | 12551 |
| TIBS033 | 32130 |
| TIBS034 | 30974 |
| TIBS035 | 25491 |
| TIBS036 | 34370 |
| TIBS037 | 32591 |
| TIBS038 | 32689 |
| TIBS039 | 29429 |
| TIBS040 | 10247 |
| TIBS041 | 38454 |
| TIBS042 | 27700 |
| **Biopsy Small Intestine** |  |
| TIBS001 | 672 |
| TIBS004 | 2970 |
| TIBS008 | 2274 |
| TIBS009 | 1496 |
| TIBS014 | 1256 |
| TIBS015 | 4744 |
| TIBS016 | 2790 |
| TIBS017 | 938 |
| TIBS018 | 1796 |
| TIBS020 | 1099 |
| TIBS021 | 2066 |
| TIBS023 | 4526 |
| TIBS024 | 3008 |
| TIBS025 | 5885 |
| TIBS026 | 9057 |
| TIBS027 | 22416 |
| TIBS028 | 27732 |
| TIBS029 | 26037 |
| TIBS030 | 3005 |
| TIBS032 | 7171 |
| TIBS033 | 9647 |
| TIBS034 | 1481 |
| TIBS036 | 11656 |
| TIBS037 | 1653 |
| TIBS038 | 29555 |
| TIBS039 | 894 |
| TIBS040 | 6279 |
| TIBS041 | 1564 |
| **Feces** |  |
| TIBS001 | 31073 |
| TIBS002 | 32869 |
| TIBS004 | 37725 |
| TIBS005 | 32871 |
| TIBS006 | 33682 |
| TIBS009 | 36269 |
| TIBS010 | 33989 |
| TIBS013 | 31788 |
| TIBS014 | 32869 |
| TIBS015 | 39839 |
| TIBS016 | 37136 |
| TIBS017 | 35260 |
| TIBS018 | 37903 |
| TIBS018 | 51576 |
| TIBS019 | 39240 |
| TIBS020 | 36333 |
| TIBS021 | 40776 |
| TIBS023 | 47177 |
| TIBS025 | 46432 |
| TIBS026 | 40288 |
| TIBS027 | 58153 |
| TIBS028 | 44137 |
| TIBS029 | 38309 |
| TIBS030 | 12204 |
| TIBS032 | 31038 |
| TIBS033 | 44115 |
| TIBS034 | 42686 |
| TIBS035 | 39264 |
| TIBS036 | 55737 |
| TIBS037 | 46926 |
| TIBS038 | 48578 |
| TIBS039 | 39448 |
| TIBS040 | 43526 |
| TIBS041 | 46143 |
| TIBS042 | 42196 |

Table S3. *Relative abundance of the bacteria that differed significantly between feces, small intestinal biopsies and colonic biopsies, respectively, in IBS patients and healthy subjects. Underlined bacteria differed significantly between the sample types of both IBS patients and healthy subjects.*

| **IBS** |  |  |  |  |  |  |
| --- | --- | --- | --- | --- | --- | --- |
| **Bacteria** | **Feces (F, n=16)** | **Small intestinal mucosa (SIM, n=12)** | **Colonic mucosa (CM, n=16)** | ***p*-value F vs SIM** | ***p*-value F vs CM** | ***p*-value SIM vs CM** |
| U. Bacteria | 0.9 (0.6-1.8) | 0.1 (4.2*10^-3^-0.6) | 3.0 (2.0-5.0) | < 0.05 | < 0.05 | < 0.0001 |
| *Leifsonia* | 0.0 (0.0-0.0) | 0.6 (0.4-1.8) | 0.04 (0.02-0.11) | < 0.0001 | < 0.01 | < 0.05 |
| *Alloprevotella* | 0.0 (0.0-0.0) | 0.4 (0.1-1.8) | 1.8 * 10^-3^ (0.0-0.05) | < 0.01 | n.s. | < 0.01 |
| *Prevotella* | 0.0 (0.0-0.0) | 0.6 (0.2-2.1) | 3.7*10^-3^ (0.0-0.02) | < 0.001 | n.s. | < 0.001 |
| *Prevotella 9* | 2.9*10^-3^ (0.0-9.5*10^-3^) | 0.05 (2.9*10^-3^-0.62) | 0.5 (0.06-2.76) | n.s. | n.s. | n.s. |
| *Alistipes* | 2.1 (0.7-3.2) | 1.9*10^-3^ (0.0-0.1) | 1.2 (0.4-1.8) | n.s. | < 0.01 | n.s. |
| *Gemella* | 0.0 (0.0-0.0) | 0.5 (0.2-0.9) | 0.0 (0.0-2.1*10^-3^) | < 0.001 | n.s. | < 0.001 |
| U. Lactobacillales | 0.0 (0.0-2.4*10^-3^) | 0.6 (0.1-1.4) | 0.0 (0.0-3.5*10^-3^) | < 0.0001 | n.s. | < 0.0001 |
| *Streptococcus* | 0.07 (0.02-024) | 8.6 (3.0-15.2) | 0.3 (0.2-0.4) | < 0.0001 | n.s. | < 0.01 |
| U. Clostridiales | 7.6 (4.5-9.9) | 0.3 (0.1-1.7) | 9.6 (7.1-10.8) | < 0.001 | n.s. | < 0.0001 |
| U.Clostridiaceae 1 | 0.01 (0.00-0.04) | 0.0 (0.0-0.0) | 0.11 (0.04-0.41) | n.s. | < 0.01 | < 0.0001 |
| *Anaerococcus* | 0.0 (0.0-0.0) | 0.0 (0.0-0.0) | 4.9*10^-3^ (0.00-0.02) | n.s. | < 0.01 | < 0.01 |
| *Ezakiella* | 0.0 (0.0-2.5*10^-3^) | 0.0 (0.0-0.0) | 0.01 (6.9*10^-4^-0.07) | n.s. | < 0.01 | < 0.001 |
| *Finegoldia* | 0.0 (0.0-0.0) | 0.0 (0.0-0.0) | 3.7*10^-3^ (0.00-0.02) | n.s. | < 0.001 | < 0.001 |
| *Peptoniphilus* | 0.0 (0.0-0.0) | 0.0 (0.0-0.0) | 9.6*10^-3^ (7.8*10-4-0.02) | n.s. | < 0.001 | < 0.01 |
| *Family XIII AD3011 group* | 0.04 (9.7 *10^-3^-0.15) | 0.0 (0.0-0.0) | 0.13 (0.07-0.20) | < 0.01 | n.s. | < 0.0001 |
| *Marvinbryantia* | 0.02 (3.2*10^-3^-0.03) | 0.0 (0.0-0.0) | 0.06 (0.04-0.09) | n.s. | < 0.01 | < 0.0001 |
| U. Ruminococcaceae | 7.6 (4.6-9.4) | 0.36 (0.02-1.74) | 7.6 (4.9-10.2) | < 0.001 | n.s. | < 0.0001 |
| *Butyricicoccus* | 0.3 (0.2-0.5) | 0.0 (0.0-0.2) | 0.7 (0.5-0.9) | < 0.05 | n.s. | < 0.0001 |
| *Ruminiclostridium 9* | 0.14 (0.08-0.26) | 0.0 (0.0-0.01) | 0.13 (0.08-0.30) | < 0.001 | n.s. | < 0.001 |
| *Veillonella* | 0.04 (0.02-0.09) | 6.2 (2.1-20.6) | 0.03 (0.01-0.08) | < 0.001 | n.s. | < 0.0001 |
| *Sutterella* | 0.09 (0.02-0.46) | 0.0 (0.0-0.1) | 0.8 (0.2-1.3) | n.s. | < 0.05 | < 0.0001 |
| *Massilia* | 0.0 (0.0-0.0) | 6.4 (2.1-10.5) | 0.12 (0.07-0.50) | < 0.0001 | < 0.001 | n.s. |
| U. Halomonadaceae | 0.0 (0.0-0.0) | 0.7 (0.0-1.3) | 9.2*10^-3^ (0.00-0.02) | < 0.001 | < 0.01 | n.s. |
| *Halomonas phoceae* | 0.0 (0.0-0.0) | 1.5 (0.0-4.9) | 0.03 (2.7*10^-3^-0.12) | < 0.0001 | < 0.01 | n.s. |
| *Pseudomonas* | 0.0 (0.0-3.1*10^-3^) | 12.8 (4.7-33.3) | 0.2 (0.1-0.8) | < 0.0001 | < 0.001 | < 0.05 |
| **HS** |  |  |  |  |  |  |
| **Bacteria** | **Feces (F, n=19)** | **Small intestinal mucosa (SM, n=16)** | **Colonic mucosa (CM, n=19)** | ***p*-value F vs SIM** | ***p*-value F vs CM** | ***p*-value SM vs CM** |
| U. Bacteria | 0.4 (0.3-0.7) | 0.10 (0.00-1.17) | 2.5 (1.5-3.4) | n.s. | < 0.0001 | < 0.0001 |
| *Leifsonia* | 0.0 (0.0-0.0) | 1.0 (0.4-1.2) | 0.2 (9.8*10^-3^-0.4) | < 0.0001 | < 0.001 | < 0.01 |
| *Gemella* | 0.0 (0.0-0.0) | 0.7 (0.2-1.4) | 0.0 (0.0-0.0) | < 0.001 | n.s. | < 0.0001 |
| U. Lactobacillales | 0.0 (0.0-0.0) | 0.8 (0.2-1.7) | 3.1*10^-3^ (0.0-9.7*10^-3^) | < 0.0001 | n.s. | < 0.001 |
| *Streptococcus* | 0.06 (0.01-0.14) | 10.2 (3.7-20.1) | 0.2 (0.2-0.5) | < 0.0001 | n.s. | < 0.01 |
| *Ezakiella* | 0.0 (0.0-2.3*10^-3)^ | 0.0 (0.0-0.0) | 0.01 (0.00-0.03) | n.s. | < 0.01 | < 0.001 |
| *Finegoldia* | 0.0 (0.0-0.0) | 0.0 (0.0-0.0) | 5.3*10^-3^ (0.00-0.01) | n.s. | < 0.001 | < 0.01 |
| *Peptoniphilus* | 0.0 (0.0-0.0) | 0.0 (0.0-0.0) | 3.0*10^-3^ (0.00-0.03) | n.s. | < 0.001 | < 0.05 |
| *Family XIII AD3011 group* | 0.03 (0.01-0.12) | 0.0 (0.0-0.0) | 0.14 (0.07-0.19) | < 0.05 | < 0.05 | < 0.0001 |
| *Butyricicoccus* | 0.3 (0.2-0.4) | 0.00 (0.00-0.2) | 0.7 (0.5-1.3) | < 0.05 | < 0.01 | < 0.0001 |
| *Massilia* | 0.0 (0.0-0.0) | 3.4 (1.2-9.8) | 0.10 (0.03-0.23) | < 0.0001 | < 0.001 | < 0.05 |
| *Pseudomonas* | 0.0 (0.0-3.0*10^-3^) | 9.2 (1.7-2.7) | 0.31 (0.07-1.10) | < 0.0001 | < 0.001 | < 0.01 |
| *Actinomyces* | 2.3*10-3 (0.0-6.1*10^-3^) | 1.9 (0.2-3.7) | 8.2*10^-3^ (0.0-0.3) | < 0.0001 | n.s. | < 0.0001 |
| *Rothia* | 0.0 (0.0-0.0) | 0.7 (0.2-1.4) | 0.0 (0.0-6.2*10^-3^) | < 0.0001 | n.s. | < 0.001 |
| *Atopobium* | 0.0 (0.0-0.0) | 0.31 (0.04-0.63) | 0.0 (0.0-0.0) | < 0.0001 | n.s. | < 0.0001 |
| *Odoribacter* | 0.07 (0.02-0.12) | 0.0 (0.0-0.0) | 0.10 (0.02-0.14) | < 0.0001 | n.s. | < 0.0001 |
| *Porphyromonas* | 0.0 (0.0-0.0) | 0.46 (0.08-0.75) | 0.0 (0.0-7.8*10^-3^) | < 0.0001 | n.s. | < 0.001 |
| *Prevotella corporis* | 0.0 (2.7*10^-3^-0.0) | 0.0 (0.0-0.0) | 5.5*10^-3^ (0.00-0.03) | n.s. | < 0.05 | < 0.01 |
| *Prevotella 7* | 0.0 (0.0-0.0) | 2.1 (0.2-3.3) | 0.0 (0.0-7.7*10^-3^) | < 0.0001 | n.s. | < 0.001 |
| *Murdochiella* | 0.0 (0.0-0.0) | 0.0 (0.0-0.0) | 0.00 (0.00-0.02) | n.s. | < 0.01 | < 0.05 |
| U. Lachnospiraceae | 6.1 (4.1-9.1) | 1.2 (0.5-4.4) | 14.3 (12.3-17.3) | < 0.05 | < 0.01 | < 0.0001 |
| *Coprococcus 3* | 0.12 (0.05-0.18) | 0.03 (0.00-0.20) | 0.68 (0.47-0.90) | n.s. | < 0.0001 | < 0.0001 |
| *Dorea* | 0.20 (0.14-0.56) | 0.04 (0.00-0.26) | 1.32 (0.86-2.00) | n.s. | < 0.0001 | < 0.0001 |
| *Eubacterium hallii group* | 0.05 (0.02-0.17) | 0.00 (0.00-0.02) | 0.90 (0.52-1.03) | n.s. | < 0.001 | < 0.0001 |
| *Lachnospiraceae FCS020 group* | 3.4*10^-3^ (0.0-8.7*10^-3^) | 0.0 (0.0-0.0) | 0.03 (0.02-0.06) | n.s. | < 0.01 | < 0.0001 |
| *Lachnospiraceae NK4A136 group* | 1.5 (1.0-2.3) | 0.02 (0.00-0.21) | 0.77 (0.47-1.82) | < 0.0001 | n.s. | < 0.001 |
| U. Erysipelotrichaceae | 0.07 (0.03-0.16) | 0.10 (2.8*10^-3^-0.17) | 0.42 (0.09-0.59) | n.s. | < 0.05 | < 0.05 |
| U. Fusobacteriales | 0.0 (0.0-0.0) | 0.06 (0.22-0.93) | 0.0 (0.0-0.0) | < 0.0001 | n.s. | < 0.0001 |
| *Fusobacterium* | 0.0 (0.0-0.0) | 0.5 (0.1-1.1) | 0.0 (0.0-3.1*10^-3^) | < 0.0001 | n.s. | < 0.0001 |

Abbrevations: U = Unclassfied

Table S4. *Relative abundance of the bacteria that differed significantly between IBS patients and healthy subjects.*

| **Bacteria** | **IBS (n=16)** | **HS (n=19)** | ***p*-value** |
| --- | --- | --- | --- |
| Feces |  |  |  |
| Unclassified Bacteria | 0.9 (0.6-1.8) | 0.4 (0.3-0.7) | < 0.01 |
| *Bifidobacterium* | 0.4 (0.1-3.0) | 0.6 (0.2-1.0) | n.s. |
| *Anaerostipes* | 0.3 (0.1-0.8) | 0.2 (0.1-0.4) | n.s. |
| *Coprococcus 3* | 0.20 (0.04-0.32) | 0.12 (0.05-0.18) | n.s. |
| *Eisenbergiella* | 2.4*10^-3^ (0.00-0.07) | 0.0 (0.0-4.5*10^-3^) | n.s. |
| *Eubacterium hallii group* | 0.11 (0.04-0.52) | 0.05 (0.02-0.17) | n.s. |
| *Eubacterium ventriosum group* | 0.14 (0.04-0.22) | 0.05 (0.02-0.09) | < 0.05 |
| *Lachnoclostridium* | 0.37 (0.16-1.00) | 0.07 (0.06-0.23) | < 0.01 |
| *Candidatus Soleaferrea* | 0.02 (4.2*10^-3^-0.02) | 5.2*10^-3^ (0.0-8.0*10^-3^) | < 0.01 |
| *Faecalibacterium* | 16.8 (9.9-20.1) | 23.6 (12.5-35.11) | n.s. |
| *Hydrogenoanaerobacterium* | 0.00 (0.00-2.9*10^-3^) | 0.00 (0.00-2.3*10^-3^) | n.s. |
| *Intestinimonas butyriciproducens* | 0.01 (0.00-0.04) | 2.7*10^-3^ (0.0-5.5*10^-3^) | n.s. |
| *Oscillospira* | 0.03 (2.6*10^-3^-0.10) | 0.01 (4.2*10^-3^-0.04) | n.s. |
| *Ruminiclostridium 5* | 0.10 (8.7*10^-3^-0.70) | 0.01 (2.3*10^-3^-0.05) | n.s. |
| *Ruminococcaceae UCG-010* | 0.05 (1.0*10^-3^-0.32) | 0.34 (0.09-1.05) | < 0.05 |
| *Ruminococcaceae UCG-013* | 0.49 (0.23-1.29) | 0.35 (0.15-0.71) | n.s. |
| *Holdemania massiliensis* | 0.00 (0.00-0.01) | 0.00 (0.00-0.00) | n.s. |
| Unclassified Saccharibacteria | 1.3*10^-3^ (0.00-5.2*10^-3^) | 0.00 (0.00-0.00) | < 0.05 |
| Colonic Biopsies | **IBS (n=16)** | **HS (n=16)** |  |
| *Odoribacter* | 0.04 (7.7*10^-3^-0.06) | 0.10 (0.03-0.14) | n.s. |
| Unclassified Prevotellaceae | 0.11 (0.07-1.13) | 0.01 (0.00-0.24) | < 0.05 |
| *Staphylococcus* | 0.00 (0.00-0.00) | 3.2*10^-3^ (0.00-0.01) | < 0.05 |
| *Christensenellaceae R-7 group* | 0.76 (0.44-1.29) | 1.55 (0.73-2.96) | < 0.05 |
| *Coprococcus 1* | 0.02 (7.5*10^-3^-0.04) | 0.06 (0.03-0.10) | < 0.01 |
| *Coprococcus 3* | 0.35 (0.09-0.59) | 0.68 (0.47-0.90) | < 0.01 |
| *Eisenbergiella* | 0.00 (0.00-0.01) | 0.00 (0.00-0.00) | n.s. |
| *Lachnoclostridium* | 0.34 (0.18-0.55) | 0.18 (0.08-0.25) | < 0.01 |
| *Lachnospira* | 0.12 (0.04-0.23) | 0.24 (0.12-0.68) | < 0.05 |
| *Anaerofilum* | 0.00 (0.00-0.00) | 0.0 (0.0-8.2*10^-3^) | n.s. |
| *Candidatus Soleaferrea* | 0.01 (8.3*10^-3^-0.02) | 5.4*10^-3^ (0.00-8.2*10^-3^) | < 0.001 |
| *Oscillibacter* | 0.00 (0.00-5.1*10^-3^) | 6.2*10^-3^ (0.00-0.02) | < 0.05 |
| *Ruminococcaceae UCG-003* | 0.04 (0.02-0.16) | 0.10 (0.06-0.16) | n.s. |
| *Ruminococcaceae UCG-010* | 0.13 (0.03-0.22) | 0.15 (0.08-0.82) | n.s. |
| *Faecalitalea* | 4.8*10^-3^ (0.00-0.03) | 0.00 (0.00-2.7*10^-3^) | < 0.05 |
| *Dialister* | 1.39 (0.08-2.55) | 0.83 (0.21-1.30) | n.s. |
| *Victivallis* | 0.00 (0.00-5.1*10^-3^) | 7.7*10^-3^ (0.00-0.02) | < 0.05 |
| Small Intestnal Biopsies | **IBS (n=12)** | **HS (n=19)** |  |
| *Rothia* | 0.29 (0.00-0.58) | 0.67 (0.19-1.39) | n.s. |
| *Atopobium* | 0.07 (0.00-1.15) | 0.31 (0.05-0.63) | n.s. |
| *Odoribacter* | 0.00 (0.00-0.00) | 0.00 (0.00-0.00) | n.s. |
| *Porphyromonas* | 0.14 (0.00-0.22) | 0.46 (0.08-0.75) | < 0.05 |
| *Paraprevotella* | 0.00 (0.00-0.00) | 0.00 (0.00-0.00) | n.s. |
| *Streptococcus* | 8.6 (3.0-15.2) | 10.2 (0.37-20.1) | n.s. |
| Unclassified Clostridiaceae 1 | 0.00 (0.00-0.00) | 1.8*10^-3^ (0.00-0.04) | < 0.05 |
| *Anaerostipes* | 7.0*10^-3^ (0.00-0.07) | 0.07 (0.00-0.31) | n.s. |
| *Lachnoanaerobaculum* | 5.1*10^-3^ (0.00-0.53) | 0.12 (8.6*10^-3^-0.30) | n.s. |
